# Tandem mass tag-based quantitative proteomics analysis reveals the new regulatory mechanism of progranulin in influenza virus infection

**DOI:** 10.3389/fmicb.2022.1090851

**Published:** 2023-01-12

**Authors:** Haoning Li, Yuying Zhang, Chengye Li, Peng Ning, Hailiang Sun, Fanhua Wei

**Affiliations:** ^1^College of Agriculture, Ningxia University, Yinchuan, China; ^2^School of Biological Science and Technology, University of Jinan, Jinan, China; ^3^College of Veterinary Medicine, South China Agricultural University, Guangzhou, China

**Keywords:** influenza virus, PGRN, multiplex tandem mass tag, proteome, lysosome

## Abstract

Progranulin (PGRN) plays an important role in influenza virus infection. To gain insight into the potential molecular mechanisms by which PGRN regulates influenza viral replication, proteomic analyzes of whole mouse lung tissue from wild-type (WT) versus (*vs*) PGRN knockout (KO) mice were performed to identify proteins regulated by the absence vs. presence of PGRN. Our results revealed that PGRN regulated the differential expression of ALOX15, CD14, CD5L, and FCER1g, etc., and also affected the lysosomal activity in influenza virus infection. Collectively these findings provide a panoramic view of proteomic changes resulting from loss of PGRN and thereby shedding light on the functions of PGRN in influenza virus infection.

## Introduction

Influenza virus belongs to the family of Orthomyxoviridae and is one of the leading causes of respiratory tract infection that results in approximately 290,000–650,000 deaths each year worldwide. Up to know, there are four distinct types of influenza virus have been reported, named as A, B, C and D. Influenza A and B viruses (IAV and IBV) are the predominant cause of human infection, whereas influenza C virus (ICV) is the cause of sporadic disease in children. Influenza D virus infects cattle. IAVs are further divided into subtypes on the basis of the hemagglutinin (HA), neuraminidase (NA) on the surface of the virus. Currently, there are 18 known HA subtypes (H1 to H18) and 11 known NA subtypes (N1 to N11) (Tong et al., 2013).

IAV is characterized as a single-stranded negative-sense RNA genome, consisting of eight segments encoding 10 core polyproteins: the glycoproteins HA and NA, nucleoprotein (NP), the matrix protein M1, the M2 ion channel, the RNA-dependent RNA polymerase (RdRp) subunits polymerase basic 1 (PB1), polymerase basic 2 (PB2), and polymerase acidic (PA), the nonstructural protein NS1, and the nuclear export protein (NEP; also known as NS2). Owing to its RNA genome, influenza virus utilizes host factors for its replication (Peacock et al., 2019). Hence, the development of effective interventions targeting host cell factors that are required by IAV for replication or persistence cellular proteins or functions is a promising antiviral strategy (Eisfeld et al., 2015; Kaufmann et al., 2018).

The innate immune system is the first line of defense against IAV infection through recognition of influenza viral RNA (vRNA) by toll-like receptors (TLRs), retinoic acid-inducible gene-I (RIG-I) receptors (RLRs) and nucleotide oligomerization domain (NOD)-like receptors (NLRs) (Takeuchi and Akira, 2009; Blasius and Beutler, 2010; Pang and Iwasaki, 2011). This process induces the expression of type I interferons (IFN-I) and pro-inflammatory cytokines by activation of IRF3/7 and NF-κB transcriptional factors (Loo and Gale Jr., 2011). IFN-I binds to the IFN-α/β receptor (IFNAR) on the infected cell or neighboring cells, results in recruitment and activation of the signal transducer and activator of transcription 1 (STAT1) and STAT2 (Schindler et al., 2007; Schulz and Mossman, 2016), and induces expression of interferon-stimulated genes (ISGs) by forming the IFN-stimulated gene factor 3 (ISGF3) and then translocating into the nucleus that establish the cellular antiviral state (Schulz and Mossman, 2016). Not surprisingly, IAV has developed many efficient mechanisms to counteract IFN-I production and to antagonize its effects (Li et al., 2020). Furthermore, avian influenza strains explore additional adaptations to counteract mammalian antiviral immune pathways. For example, substitution of the avian-signature glutamate at position 627 and aspartate 701 to mammalian-signature 627 K and 701 N are common in zoonotic and human-adapted strains (Subbarao et al., 1993; Czudai-Matwich et al., 2014), which masks nucleocapsid inhibition by the pathogen sensor RIG-I (Weber et al., 2015).

Progranulin (PGRN), also known as granulin-epithelin precursor, PC-cell-derived growth factor, and acrogranin, consists of seven-and-a-half cysteine-rich motif with a unique bead-like structure (Hrabal et al., 1996; Chitramuthu et al., 2017). PGRN plays a crucial role in inflammatory response (Zhu et al., 2002; He et al., 2003; Kessenbrock et al., 2008; Tang et al., 2011; Lu et al., 2021; Liu et al., 2022), host defense (Yin et al., 2010; Wang et al., 2019), frontotemporal dementia (Baker et al., 2006; Cruts et al., 2006), and lysosomal storage disease (Jian et al., 2016). PGRN is induced in human cells and in mice lung samples after infection with influenza virus (Brandes et al., 2013; Luo et al., 2019; Wei et al., 2019). Our previous data suggest that influenza virus-inducing PGRN negatively regulated IFN-I production by inhibiting NF-κB and IRF3 activation and identify a PGRN-mediated IFN-I evasion pathway exploited by influenza virus (Wei et al., 2019). In addition, PGRN deficiency leads to reduced influenza viral replication and PGRN-deficient mice sustain a lesser degree of lung inflammatory response after influenza infection (Wei et al., 2019). These facts mean that PGRN plays a key role in influenza virus infection, however, the precise role of PGRN in influenza virus infection has not been elucidated.

In the present study, we utilized a proteomic approach to study the effect of knocking out PGRN on global protein expression in the influenza virus-infected mice lung tissue. Our findings provide valuable information for understanding the role of PGRN in influenza virus infection, which may contribute to further elucidating the pathogenesis of influenza virus and the development of effective treatments for influenza virus.

## Materials and methods

### Animal experiments

Animal experiments were performed as described (Wei et al., 2019). 6-to 8-week-old wild-type (WT) and PGRN KO mice (*n* = 2 per group) were mock-or infected intranasally with a low dose (100 TCID_50_) of influenza A/Puerto Rico/8/1934 (PR8) virus in 50 μl Phosphate Buffered Saline (PBS) after anesthesia. Lung samples were collected, weighted, frozen in liquid nitrogen and stored at-80°C for further sample processing.

### Protein extraction

The samples were grinded by liquid nitrogen and lysed with lysis buffer containing 8 M urea and 1% protease inhibitor cocktail, followed by sonication 3 times on ice using a high-intensity ultrasonic processor (Scientz). The remaining debris was removed by centrifugation at 12,000 × g for 10 min at 4°C. Finally, sample supernatants were collected and protein concentration was determined by BCA kit (Beyotime Biotechnology) according to the manufacturer’s instructions.

### Trypsin digestion

For digestion, sample supernatants were reduced with 5 mM dithiothreitol for 30 min at 56°C and were alkylated with 11 mM iodoacetamide for 15 min at room temperature (RT). The protein samples were then diluted by adding 100 mM TEAB (Sigma) and trypsin was added at 1:50 ratio for the first digestion overnight and 1:100 for the second digestion for 4 h.

### Tandem mass tag labeling and peptide fractionation

After trypsin digestion, 100 mg of peptides were desalted by Strata™ X-C SPE column (Phenomenex) and reconstituted in 0.5 M TEAB and processed according to the manufacturer’s protocol for TMT kit. The labeled peptides were then incubated for 2 h at RT and mixed in equal amounts, desalted and dried by vacuum centrifugation. The dried peptides were fractionated using a High-pH reversed-phase column (Thermo Fisher Scientific). Briefly, peptides were separated with a gradient of 8 to 32% acetonitrile (pH 9.0) over 60 min into 60 fractions. Then, the peptides were combined into 6 fractions and dried by vacuum centrifuging.

### LC–MS/MS analysis

The tryptic peptides were dissolved in 0.1% formic acid (solvent A), loaded onto a reversed-phase analytical column (15 cm length, 75 μm i.d.). The gradient was comprised of an increased concentration from 6 to 23% solvent B (0.1% formic acid in 85% acetonitrile) over 26 min, 23 to 35% solution for 8 min, 80% solution for 3 min, and hold at 80% solution for 3 min. The EASY-nLC 1,000 UPLC system was used for separation at a constant flow rate of 400 nl/min.

The peptide fraction was separated by chromatography and analyzed on a Q-Exactive™ Plus (Thermo) by tandem mass spectrometry. The electrospray voltage applied was 2.0 kV. The scan range was from 350 to 1800 mass-charge ratio (m/z) for full scan. The first-order mass spectrum resolution was 70,000 at an m/z of 200. Peptides were then selected for MS/MS using NCE setting as 28 and the second-order mass spectrum resolution was 17,500 at an m/z of 200. A data-dependent procedure that alternated between one MS scan followed by 20 MS/MS scans with 15.0 s dynamic exclusion. Automatic gain control (AGC) was set at 5e4 and was used to prevent overfilling of the orbitrap.

### Database search

The resulting MS/MS data were processed using Maxquant search engine (v.1.5.2.8). Tandem mass spectra were searched against mouse UniProt database concatenated with reverse decoy database. The mass tolerance for precursor ions was set as 20 ppm in the first search and 5 ppm in the main search, and the mass tolerance for fragments was set as 0.02 Da. Carbamidomethyl on Cys was specified as fixed modification and acetylation modification and oxidation on Met were specified as variable modifications. False positive rate (FDR) identified by PSM was set to 1% and minimum score for modified peptides was set >40.

### Statistical analysis

The heat maps using Cluster 3.0 and Java Treeview 1.1.6r4, and the volcano plots and Venn graphs and ROC using R language. The protein expression level difference among the samples was determined by student’s t-test. The *p* value <0.05 was identified as significantly DEPs, and STRING software (version 11.0) was used to perform the protein–protein interaction (PPI) analysis. All interactions that had a confidence score > 0.7 (high confidence) were fetched. Interaction network form STRING was visualized in R package “networkD3.”

## Results

### Overview of lung proteome data analysis

**Figure 1:**
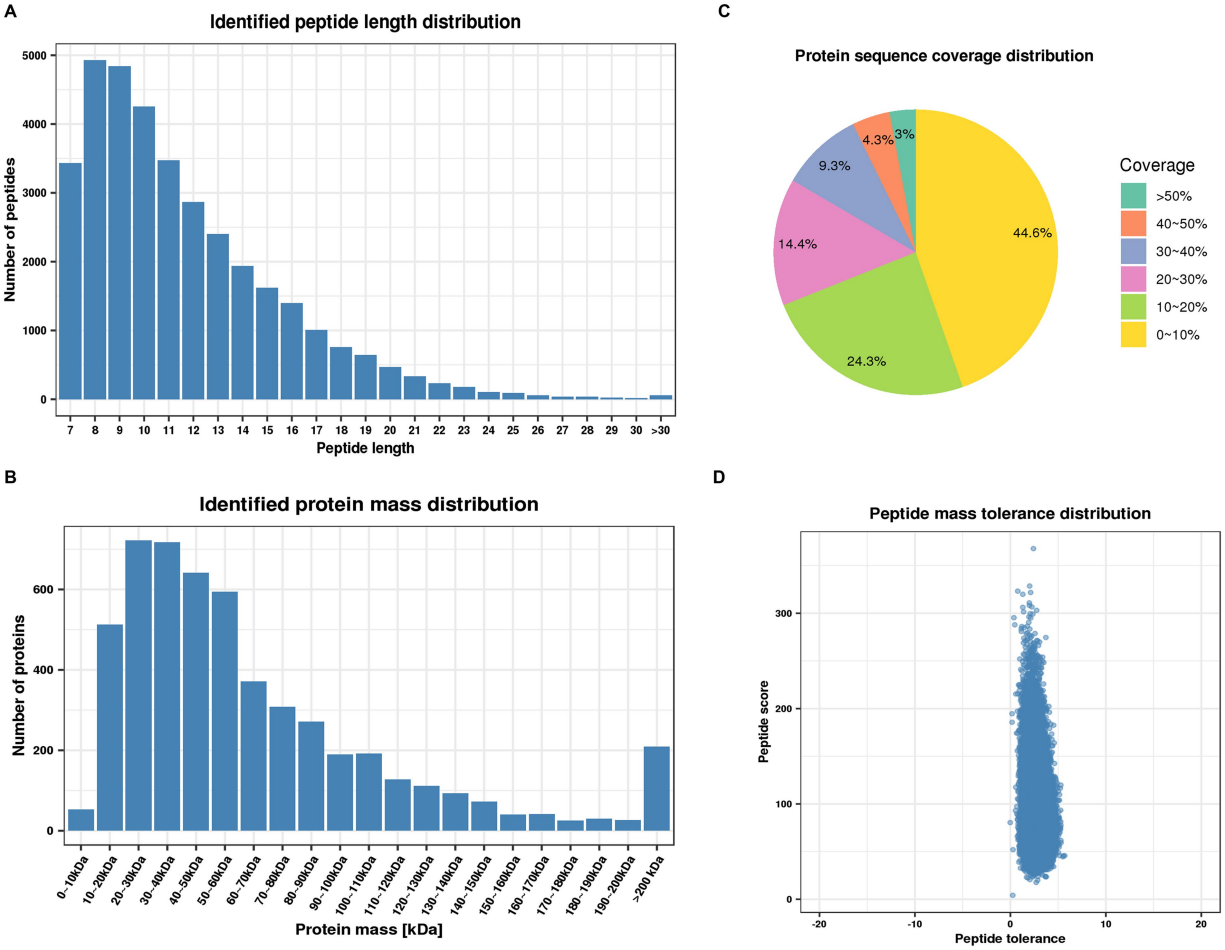
Quality control validation of mass spectrometry (MS) data. **(A)** Length distribution of peptides identified by mass spectrometry. **(B)** Molecular weight distribution of identified proteins. **(C)** Distribution of identified proteins coverage. **(D)** Distribution of mass accuracy for mass spectrometry.

To further investigate the mechanism underlying the role of PGRN in influenza virus infection, the global cellular protein expression profiles of lung tissues infected with PR8 virus at 0 day post-infection (dpi) and 3 dpi was analyzed by the multiplexed tandem mass tag (TMT) method which has been widely applied for investigating the potent antiviral agents and different signaling pathways (Yang et al., 2019). The results demonstrated that most of the peptides were distributed in the range of 7 to 20 amino acids, which is in accordance with trypsin enzymatic digestion and HCD fragmentation assays, indicating that these samples met the required standard (Figure 1A). Protein molecular weight greater than 10 kD were more uniformly distributed, indicating that no significant bias in molecular weight was generated during samples preparation (Figure 1B). The coverage of most proteins was below 20%, which is required for preferentially preferential scan of mass spectra (Figure 1C). Furthermore, the majority of the spectra had a first-order mass error of 10 ppm or less, which is consistent with the high precision characteristics of mass spectrometry (Figure 1D).

**Figure 2:**
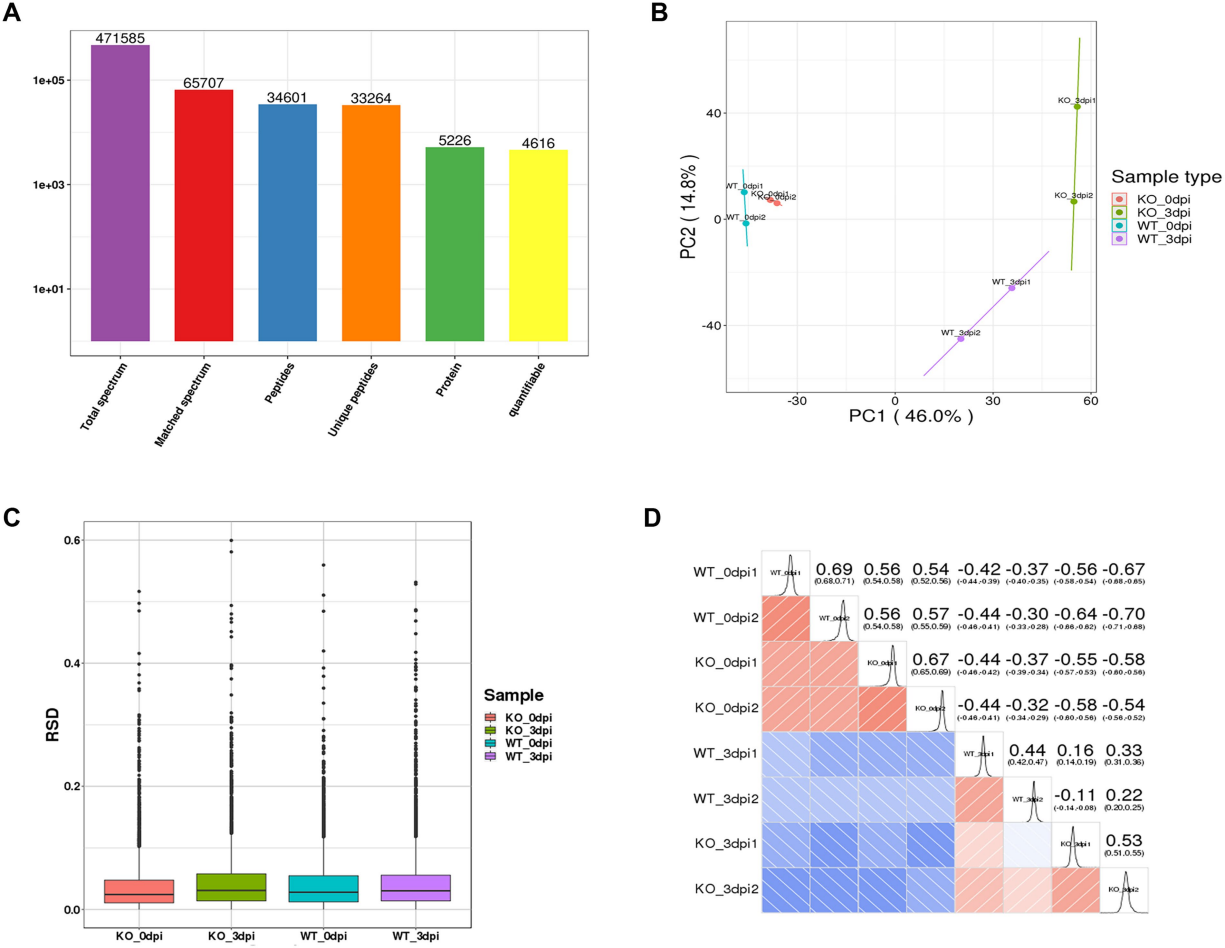
General overview of protein analysis. **(A)** Summary of MS spectrum database search analysis. **(B)** Plot results of principal component analysis (PCA) for all protein samples. **(C)** Relative standard deviation (RSD) results of all proteins samples between two replicates. **(D)** Heat map of Pearson correlation coefficient between all protein samples. The closer to –1 represents the negative correlation, the closer to 1 represents the positive correlation, and the closer to 0 represents no correlation.

After quality validation, the analysis of the proteome of lung samples from PR8 virus-infected WT and KO mice identified 5,226 distinct proteins, including 4,616 proteins that were quantified (Figure 2A). In addition, we observe high similarity in expression patterns within two biological repeats and low similarity between PR8 virus-infected WT and KO mice by three statistical analysis methods: principal component analysis (Figure 2B), relative standard deviation (Figure 2C), and Pearson correlation (Figure 2D).

### Protein differential expression analysis

Significantly differentially expressed proteins (DEPs) were determined by fold-change ratios >1.2 and *p* < 0.05 (Unwin et al., 2010). Among these, the KO group displayed significantly altered expression levels compared with WT control at 0 dpi and 3 dpi, respectively, including 113 up-regulated proteins and 73 down-regulated proteins at 0 dpi, and 259 up-regulated proteins and 113 down-regulated proteins at 3 dpi (Supplementary Table S1). Meanwhile, PR8 virus infection results in increased expression of 514 proteins and decreased expression of 420 proteins in PR8 virus-infected WT mice lung samples at 3 dpi compared with the WT control at 0 dpi. And PR8 virus infection leads to significantly altered expression levels of increased expression of 644 up-regulated proteins and 503 down-regulated proteins in PR8 virus-infected KO mice lung samples at 3 dpi compared with the KO control at 0 dpi (Supplementary Table S2).

### Subcellular localization and functional annotation of DEPs in PR8 virus-infected WT and KO mice lung tissues

**Figure 3:**
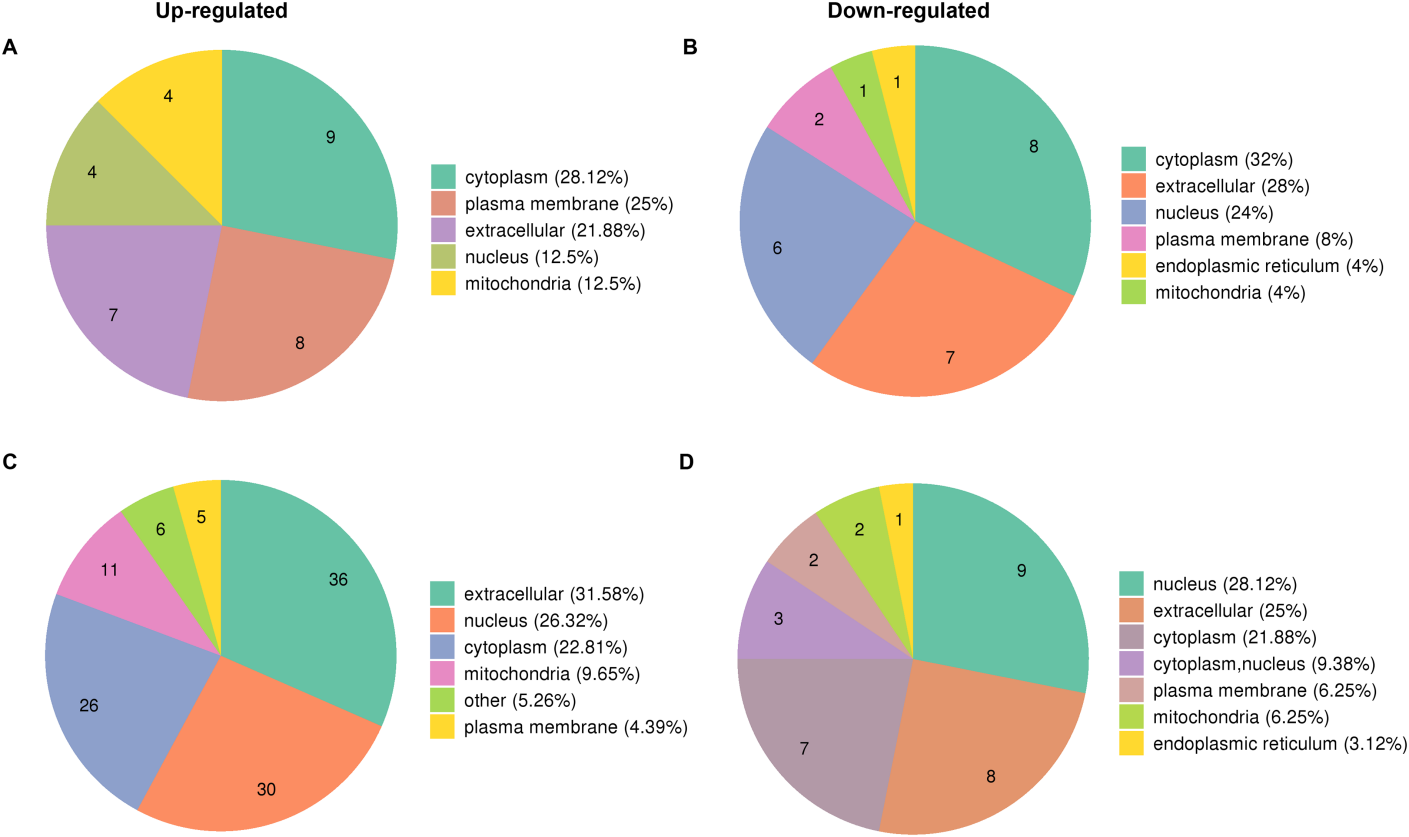
Subcellular localization of DEPs proteins in KO-d0 vs. WT-d0 and KO-d3 vs. WT-d3 groups after influenza virus infection. **(A)** The distribution of up-regulated proteins in KO-d0 vs. WT-d0 group at 0 dpi. **(B)** The distribution of down-regulated proteins in KO-d0 vs. WT-d0 group at 0 dpi. **(C)** The distribution of up-regulated proteins in KO-d3 vs. WT-d3 group at 3 dpi. **(D)** The distribution of down-regulated proteins in KO-d3 vs. WT-d3 group at 3 dpi.

The proteome profiles of PR8 virus-infected lung tissues from WT and KO mice and mock control were compared to determine the effects of PGRN virus on influenza viral replication. We predicted the subcellular localization of quantified DEPs in KO-d0 vs. WT-d0 and KO-d3 vs. WT-d3 groups after PR8 virus infection and found that most up-regulated proteins were distributed in cytosol, plasma membrane and extracellular (Figure 3A) and down-regulated proteins were distributed in cytosol, extracellular and nucleus (Figure 3B) in KO-d0 vs. WT-d0 group at 0 dpi. Upon influenza virus infection, approximately 31.58% of extracellular proteins, 26.32% of nuclear proteins and 22.81% of cytosolic proteins were up-regulated in KO-d3 vs. WT-d3 group at 3 dpi (Figure 3C). Meanwhile, approximately 28.12% of nuclear proteins, 25% of extracellular proteins and 21.88% of cytosolic proteins were down-regulated in KO-d3 vs. WT-d3 group at 3 dpi (Figure 3D). By contrast, the up-regulated proteins were distributed in extracellular, nucleus and cytosol (Supplementary Figure S1A) and down-regulated proteins were distributed in cytosol, nucleus and mitochondria (Supplementary Figure S1B) in both WT-d3 vs. WT-d0 and KO-d3 vs. KO-d0 groups after PR8 virus infection.

**Figure 4:**
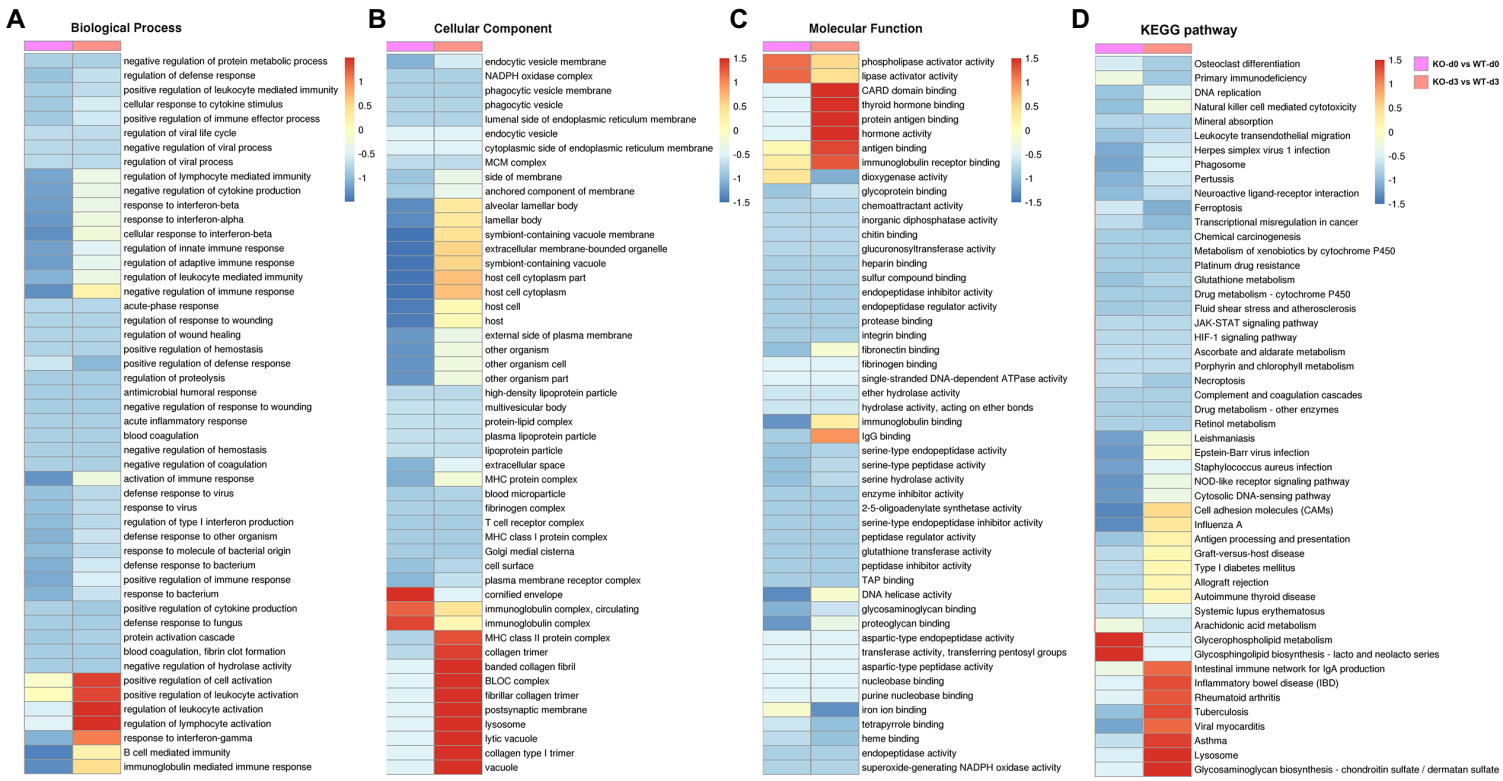
Enrichment analysis of identified DEPs in KO-d0 vs. WT-d0 and KO-d3 vs. WT-d3 groups after influenza virus infection. Classification of these proteins in different categories based on biological process **(A)**, cellular component **(B)** and molecular function **(C)**. **(D)** KEGG enrichment analysis of the DEPs in WT and KO mice lung tissues after influenza virus infection at 0 dpi and 3 dpi, respectively.

To elucidate the potential role of PGRN on influenza viral replication, gene ontology (GO) analyzes of the proteins upregulated and downregulated by PR8 virus were performed to map the genes involved in different biological processes, molecular functions, and cellular components. GO enrichment analysis showed that down-regulated DEPs of KO-d0 vs. WT-d0 group were enriched in “cellular response to interferon-beta,” “negative regulation of immune response,” “response to interferon-alpha,” and “activation of immune response,” etc. (Figure 4A). In KO-d3 vs. WT-d3 group, the up-regulated DEPs were mainly enriched in “regulation of leukocyte activation,” “regulation of lymphocyte activation,” “positive regulation of leukocyte activation,” and “response to interferon-gamma,” etc. (Figure 4A). Moreover, the down-regulated DEPs were enriched in “positive regulation of defense response,” “regulation of viral process,” etc. (Figure 4A). As for the biological cellular component enrichment, the most significantly enriched cellular components for influenza virus-infected WT and KO mice lung samples were the cornified envelope and immunoglobulin complex at 0 dpi, and MHC class II protein complex, BLOC complex, lysosome and vacuole at 3 dpi (Figure 4B). In addition, the GO terms of DEPs influenza virus-infected WT and KO mice lung samples were strongly represented by “phospholipase activator activity” and “lipase activator activity” at 0 dpi, and “CARD domain binding,” “protein antigen binding,” “hormone activity,” and “immunoglobulin receptor activity” at 3 dpi, etc. in molecular functions (Figure 4C). These results showed differences between WT and KO mice lung tissues after influenza virus infection, and further analyzes of these regulated genes may shed light on the antiviral mechanism of PGRN.

To obtain more information about the biological pathways in which the DEPs may be involved, we performed KEGG enrichment analysis of differential proteins in WT and KO mice lung tissues after influenza virus infection. The differential proteins in KO-d3 vs. WT-d3 group were mainly enriched in the “Tuberculosis,” “Viral myocarditis,” “Lysosome,” and “Glycosaminoglycan biosynthesis” etc. (Figure 4D). As for KO-d0 vs. WT-d0 group, the largest four groups were “Cell adhesion molecules (CAMs),” “Influenza A,” “Glycerophospholipid metabolism,” and “Glycosphingolipid biosynthesis-lacto and neolacto series” (Figure 4D).

**Figure 5:**
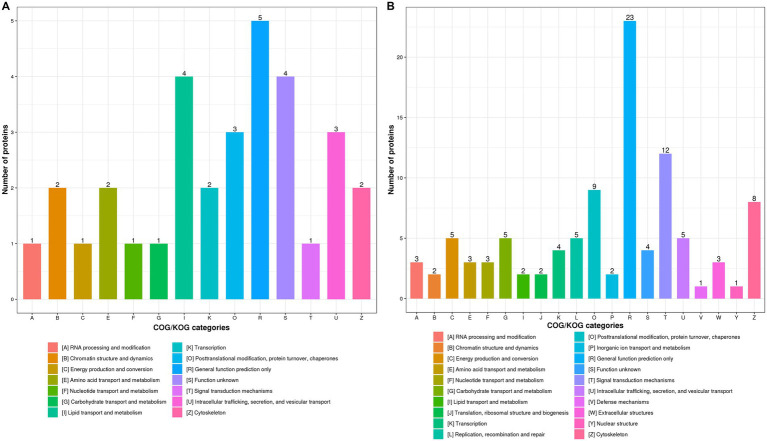
COG/KOG functional classification distribution maps of DEPS in WT and KO mice lung tissues infected with PR8 virus at 0 dpi **(A)** and 3 dpi **(B)**, respectively.

It has been demonstrated that host protein synthesis and processing machineries can be hijacked and usurped by virus to synthesize, modify and transport viral proteins and therefore facilitate viral replication (Lum and Cristea, 2016; Rodrigo et al., 2017; Coombs, 2020). Not surprisingly, influenza virus infection led to the significantly increased expression of most DEPs in “Posttranslational modification, protein turnover, chaperones” and “Signal transduction mechanisms” in WT (Supplementary Figure S2A) and KO mice lung tissues (Supplementary Figure S2B) at 3 dpi as compared with uninfected controls. We found that expression profiles of DEPs associated with “Lipid transport and metabolism” was significantly increased in KO-d0 vs. WT-d0 group at 0 dpi (Figure 5A). However, the expression of DEPs related to “Posttranslational modification, protein turnover, chaperones” and “Intracellular trafficking, secretion, and vesicular transport” was significantly increased in KO-d3 vs. WT-d3 group at 3 dpi (Figure 5B).

### Protein interaction network analysis

**Figure 6:**
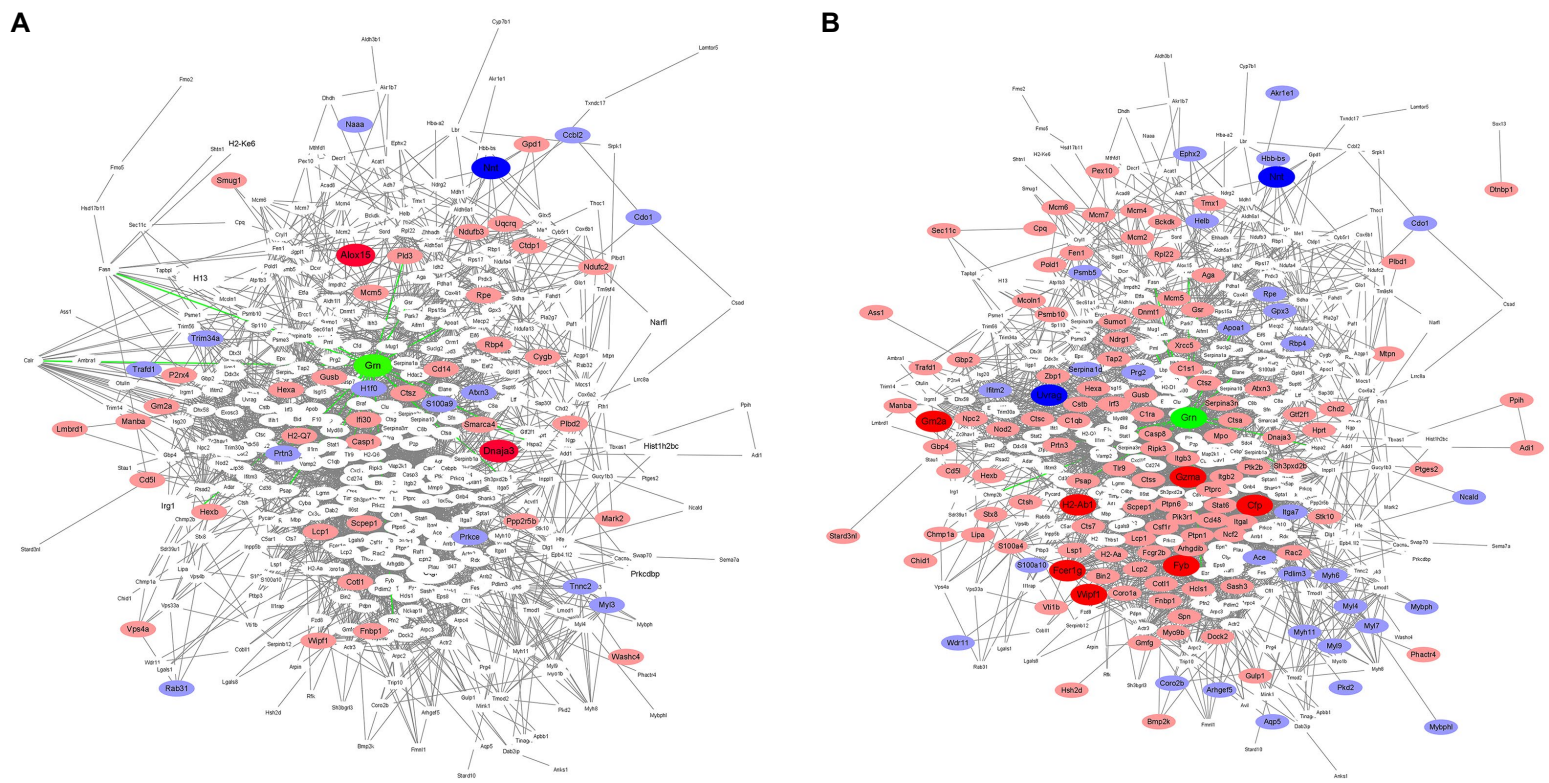
Protein interactions network. In the figure, green color indicates PGRN. Red color represents up-regulated proteins and blue indicates down-regulated proteins. Dark color represents the extremely significant changes, light color indicates the significant changes, and white represents no significant changes. **(A)** The protein–protein interactions of the DEPs in KO-d0 vs. WT-d0 group are analyzed by the STRING software. **(B)** The protein–protein interactions of the DEPs in KO-d3 vs. WT-d3 group after influenza virus infection are analyzed by the STRING software.

To further describe possible relationships between PGRN and the DEPs in influenza virus-infected WT and KO mice lung tissues at 0 dpi and 3dpi, we analyzed the protein–protein interaction network using the STRING software. We first compared the 18 common DEPs proteins-interacted with PGRN in KO-d0 vs. WT-d0 and KO-d3 vs. WT-d3 groups, including PRTN3 (Proteinase 3), GSR (Glutathione-disulfide reductase), PKM (Pyruvate kinase M1/2), HSPA2 (Heat shock protein family A (Hsp70) member 2), PFN2 (Profilin 2), STAT1 (Signal transducer and activator of transcription 1), PLD3 (Phospholipase D family member 3), PML (PML nuclear body scaffold), TLR9 (Toll-like receptor 9), PSAP (Prosaposin), AIF1 (Allograft inflammatory factor 1), APOA1 (Apolipoprotein A1), CALR (Calreticulin), CAPZA1 (Capping actin protein of muscle Z-line subunit alpha 1), CHMP2b (Charged multivesicular body protein 2B), CTSA (Cathepsin A), ELANE (Elastase, neutrophil expressed), and FASN (Fatty acid synthase; Figures 6A,B). Among them, PRTN3 was significantly down-regulated and PLD3 was significantly up-regulated in KO-d0 vs. WT-d0 group (Figure 6A). After influenza virus infection, PRTN3 was significantly increased in KO mice lung tissues at 3 dpi, while no significant changes of PLD3 expression was observed in WT and KO mice lung tissues at 3 dpi (Figure 6B).

In addition, expression of PSAP, TLR9, CTSA, and GSR was unchanged in KO-d0 vs. WT-d0 group (Figure 6A), while their expression was significantly increased in KO mice lung tissues at 3 dpi (Figure 6B). Expression of STAT1 and ELANE was not obviously changed in both KO-d0 vs. WT-d0 (Figure 6A) and KO-d3 vs. WT-d3 (Figure 6B) groups.

In addition to the 18 directly interacted proteins, we also found that significantly increased expression of ALOX15 (Arachidonate 15-lipoxygenase) and DNAJA3 (DnaJ heat shock protein family (Hsp40) member A3) and decreased expression of NNT (Nicotinamide nucleotide transhydrogenase) in KO mice lung tissues at 0 dpi (Figure 6A). Upon influenza virus infection, extremely up-regulatory expression of GZMA (Granzyme A), GM2A (GM2 ganglioside activator), H2-Ab1 (Histocompatibility 2, class II antigen A, beta 1), CFP (Complement factor properdin), FYB (FYN binding protein), FCER1g (Fc fragment of IgE receptor Ig), and WIPF1 (WAS/WASL interacting protein family member 1) and highly down-regulatory expression of UVRAG (UV radiation resistance associated) and NNT (Nicotinamide nucleotide transhydrogenase) in KO mice lung tissues at 0 dpi (Figure 6B). Furthermore, we further counted the interactions of these proteins in the network diagram and found that the top 6 number of interacted proteins were PTPRC (Protein tyrosine phosphatase receptor type C), RAC2 (Rac family small GTPase 2), CASP3 (Caspase 3), MAPK3 (Mitogen-activated protein kinase 3), STAT1 (Signal transducer and activator of transcription 1), and ITGB2 (Integrin subunit beta 2; Figures 6A,B), suggesting that these proteins are located at the core of the interaction network and play a key role in PGRN-mediated influenza virus infection.

## Discussion

Previous findings revealed that PGRN plays a key role in influenza virus infection by the IFN-I evading mechanism (Wei et al., 2019). In the present study, TMT-based quantitative proteomics was used to analyze proteins with significant differences in influenza virus-infected lung tissues from WT and PGRN KO mice. These proteins will become candidate targets for manipulation of influenza virus infection.

A total of 4,616 proteins were quantified in the lung samples from PR8 virus-infected WT and KO mice. Prior to infection with influenza virus, a total of 186 proteins were significantly different between the two groups of mice (KO-d0 vs. WT-d0 group) due to PGRN deficiency. By enrichment analysis, we localized a total of 6 differential proteins associated with host antigen presentation in this comparison group, with ALOX15 and DNAJA3 proteins are highly significantly upregulated, CD5L, CD14, and H2-Q7 are significantly upregulated, and NNT is highly significantly downregulated. After infection with influenza virus (KO-d3 vs. WT-d3 group), a total of 10 proteins involved in host antigen presentation and closely related to the 6 proteins localized before infection. H2-Ab1 and FCER1g are highly significantly upregulated, CTSS (Cathepsin S), CD5L, DNAJA3, H2-Aa, TAP2 (Transporter associated with antigen processing 2), CTSA (Cathepsin A) and MPO (Myeloperoxidase) are significantly upregulated, while NNT remained highly significantly downregulated. It has been shown that ALOX15 is highly expressed in macrophages and enhances macrophage phagocytosis (Zambuzi et al., 2021). CD14 is a leukocyte differentiation antigen present on the surface of monocytes, macrophages and other cells, and is also one of the pro-phagocytic receptors and contributes to the polarization of monocytes into M2 macrophages (Lee et al., 2020; Kim et al., 2022). Macrophages are a major source of CD5-like protein (Cd5L), which is shown to drive M2 macrophage polarization (Li et al., 2021). These results suggest that PGRN may regulates the function of antigen-presenting cells, thus making PGRN KO mice resistant to influenza virus infection.

The KEGG analysis results suggest that the differential proteins in KO-d3 vs. WT-d3 group are mainly enriched in the “Lysosome.” These findings are consistent with previous results suggest that PGRN plays a critical role in regulating lysosome function, including proteolysis, lipid degradation, and lysosomal biogenesis (Tayebi et al., 2020; Davis et al., 2021; Takahashi et al., 2021; Zhao et al., 2021; Simon et al., 2022). The enhanced activity and maturation of several cathepsins is reported in embryonic fibroblasts and aged brains from PGRN KO mice (Gotzl et al., 2018), we also observed a significantly increased expression of CTSS and CTSA in influenza virus-infected lung tissues from PGRN KO mice. Furthermore, PGRN is a regulator of autophagosome-lysosome fusion and upregulates autophagy flux *via* increased ERK1/2 kinase activity (Zhao et al., 2021; Dedert et al., 2022). It has been well-documented that influenza virus can utilize autophagy machineries to promote its replication. For example, influenza virus-induced autophagy induces the expression of proinflammatory cytokines or activation of NF-κB and p38 MAPK pathways, resulting in excessive inflammation to exacerbate acute lung injury (Pan et al., 2014; Zhang et al., 2019). Moreover, influenza virus-induced autophagy restricts interferon-β (IFN-β) production and benefits virus infection (Perot et al., 2018). In addition, influenza virus infection also affects the subcellular localization of ribosomal proteins, viral proteins and viral mRNA in autophagosomes (Becker et al., 2018). These results suggest that PGRN may affect influenza virus infection through regulating autophagy machineries.

Our subsequent research will focus on PGRN’s binding partners and differentially expressed genes regulated by PGRN in influenza virus infection. PGRN contains seven-and-a-half repeats of granulin (GRN) and exhibits anti-inflammatory activity (Tang et al., 2011; Lu et al., 2021; Shi et al., 2022) and PGRN can be cleaved by neutrophil elastase to release GRN which plays a role as a pro-inflammatory factor (Zhu et al., 2002). During influenza virus infection, neutrophil elastase activity is also increased, leading to the conversion of PGRN to GRN. To resolve this, the experiments we are conducting are designed to generate granulin-transgenic mice to identify the functions of PGRN and GRN in influenza virus infection and in virus-induced lung injury.

Collectively, the PGRN regulatory network during influenza virus infection was analyzed by proteomics to elucidate the downstream mechanisms of PGRN-mediated influenza virus infection. This study contributes to the discovery of drug targets to interfere with influenza virus infection and refines the mechanisms of PGRN-mediated immune regulation.

## Data availability statement

The datasets presented in this study can be found in online repositories. The names of the repository/repositories and accession number(s) can be found at: http://www.proteomexchange.org/, PXD038885.

## Author contributions

HL and YZ conceived, designed, and performed the experiments. CL, PN, HS, YZ, and FW analyzed the data. YZ and FW wrote the manuscript. All authors contributed to the article and approved the submitted version.

## Funding

This work was supported by the Ningxia Natural Science Foundation of China (2021AAC05006) and the National Natural Science Foundation of China (NSFC) (31972669 and 81960297).

## Conflict of interest

The authors declare that the research was conducted in the absence of any commercial or financial relationships that could be construed as a potential conflict of interest.

## Publisher’s note

All claims expressed in this article are solely those of the authors and do not necessarily represent those of their affiliated organizations, or those of the publisher, the editors and the reviewers. Any product that may be evaluated in this article, or claim that may be made by its manufacturer, is not guaranteed or endorsed by the publisher.

## Supplementary material

The Supplementary material for this article can be found online at: https://www.frontiersin.org/articles/10.3389/fmicb.2022.1090851/full#supplementary-material
