# Supplementary material for: Tandem mass tag-based quantitative proteomics analysis reveals the new regulatory mechanism of progranulin in influenza virus infection
Source: Front Microbiol. 2023 Jan 12;13:1090851. doi: 10.3389/fmicb.2022.1090851 (PMC9877624; doi:10.3389/fmicb.2022.1090851)
Supplement: Supplementary file 1 [file Table_1.DOCX]

**Table 1 Summary of differentially expressed proteins in each group**

| **Group** | **Up-regulated** | **Down-regulated** |
| --- | --- | --- |
| KO-d0 vs WT-d0 | 113.0 | 73.0 |
| KO-d3 vs KO-d0 | 644.0 | 503.0 |
| KO-d3 vs WT-d3 | 259.0 | 113.0 |
| WT-d3 vs WT-d0 | 514.0 | 420.0 |
