# Supplementary material for: Tandem mass tag-based quantitative proteomics analysis reveals the new regulatory mechanism of progranulin in influenza virus infection
Source: Front Microbiol. 2023 Jan 12;13:1090851. doi: 10.3389/fmicb.2022.1090851 (PMC9877624; doi:10.3389/fmicb.2022.1090851)
Supplement: Supplementary file 2 [file Table_2.DOCX]

**Table 2. Potential role of PGRN-interacted proteins in influenza virus infection.**

| Proteins | Function |
| --- | --- |
| PRTN3 |  |
| GSR |  |
| PKM |  |
| HSPA2 |  |
| PFN2 |  |
| STAT1 |  |
| PLD3 |  |
| PML |  |
| TLR9 |  |
| PSAP |  |
| AIF1 |  |
| APOA1 |  |
| CALR |  |
| CAPZA1  CHMP2b  CTSA  ELANE  FASN |  |
