# Supplementary figures and images for: Tandem mass tag-based quantitative proteomics analysis reveals the new regulatory mechanism of progranulin in influenza virus infection
Source: Front Microbiol. 2023 Jan 12;13:1090851. doi: 10.3389/fmicb.2022.1090851 (PMC9877624; doi:10.3389/fmicb.2022.1090851)

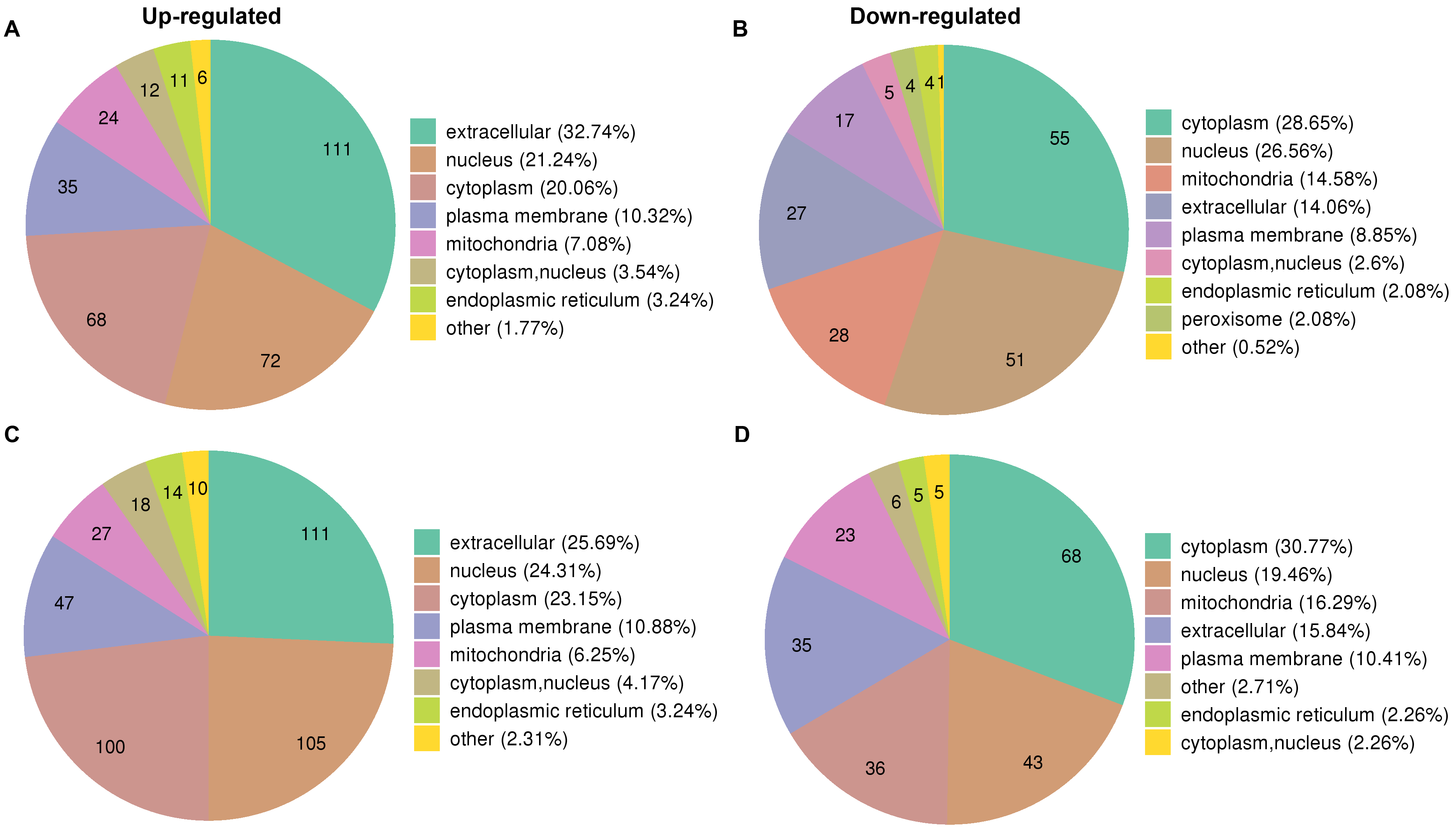

Supplement: Supplementary file 3 [file Image_1.TIF]

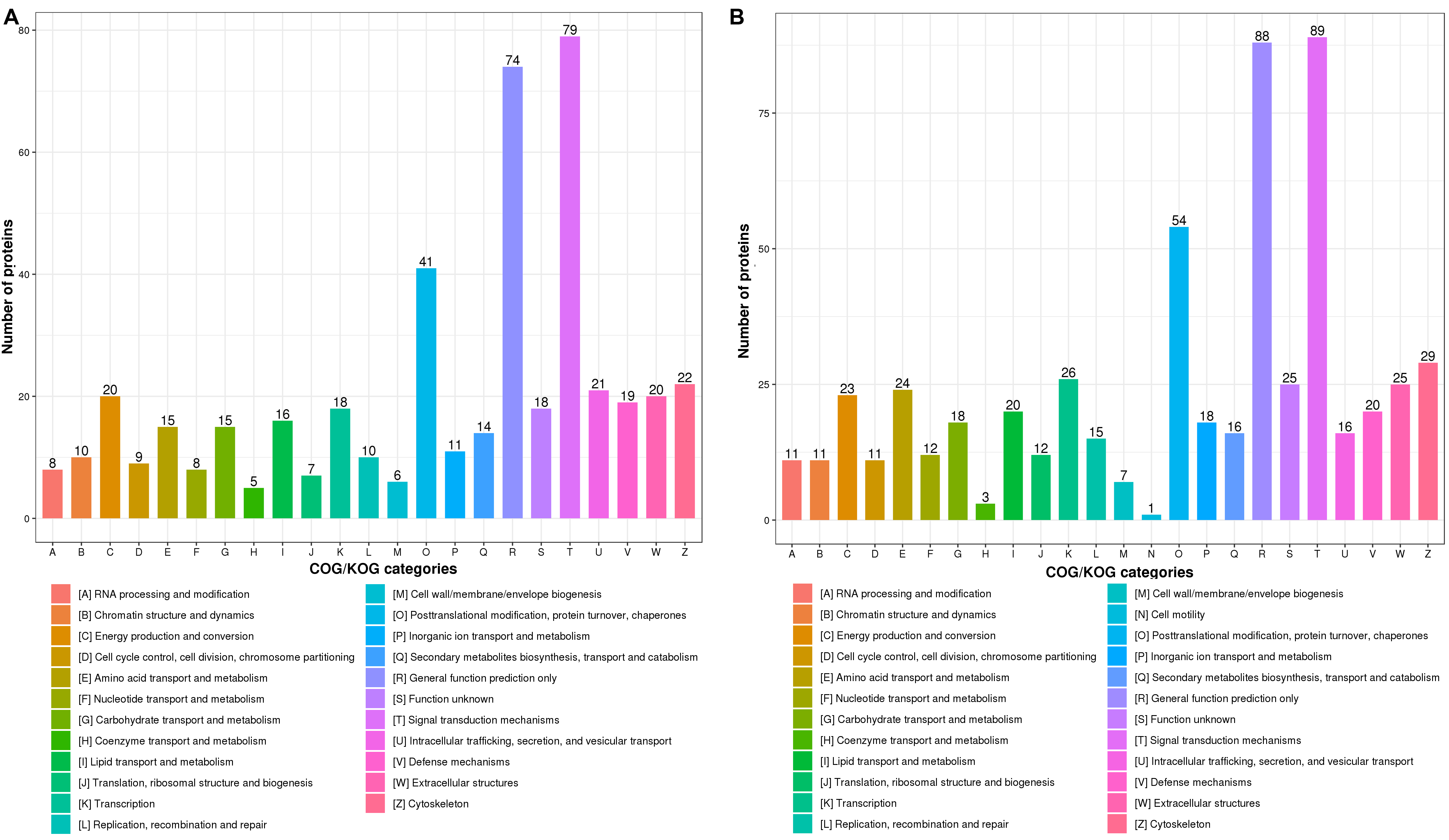

Supplement: Supplementary file 4 [file Image_2.TIF]
